# NIPT Technique Based on the Use of Long Chimeric DNA Reads

**DOI:** 10.3390/genes11060590

**Published:** 2020-05-26

**Authors:** Vera Belova, Daria Plakhina, Sergey Evfratov, Kirill Tsukanov, Gennady Khvorykh, Alexander Rakitko, Alexander Konoplyannikov, Valery Ilinsky, Denis Rebrikov, Dmitriy Korostin

**Affiliations:** 1 Center for Precision Genome Editing and Genetic Technologies for Biomedicine, Pirogov Medical University, 1 Ostrovityanova Street, 117997 Moscow, Russia; verusik.belova@gmail.com (V.B.); konoplyannikov@yandex.ru (A.K.); drebrikov@gmail.com (D.R.); 2 Genotek Ltd., 17-1-14 Nastavnicheskiy Pereulok, 105120 Moscow, Russia; daria.plahina@gmail.com (D.P.); evfratov@gmail.com (S.E.); tsukanoffkirill@gmail.com (K.T.); gennady.khvorykh@gmail.com (G.K.); rakitko@gmail.com (A.R.); valery@genotek.ru (V.I.)

**Keywords:** NIPT, chimeric DNA, cfDNA, NGS, short DNA fragments, fetal fraction, long chimeric reads

## Abstract

Non-invasive prenatal testing (NIPT) for aneuploidy on Chromosomes 21 (T21), 18 (T18) and 13 (T13) is actively used in clinical practice around the world. One of the limitations of the wider implementation of this test is the high cost of the analysis itself, as high-throughput sequencing is still relatively expensive. At the same time, there is an increasing trend in the length of reads yielded by sequencers. Since extracellular DNA is short, in the order of 140–160 bp, it is not possible to effectively use long reads. The authors used high-performance sequencing of cell-free DNA (cfDNA) libraries that went through additional stages of enzymatic fragmentation and random ligation of the resulting products to create long chimeric reads. The authors used a controlled set of samples to analyze a set of cfDNA samples from pregnant women with a high risk of fetus aneuploidy according to the results of the first trimester screening and confirmed by invasive karyotyping of the fetus using laboratory and analytical approaches developed by the authors. They evaluated the sensitivity, specificity, PPV (positive predictive value), and NPV (negative predictive value) of the results. The authors developed a technique for constructing long chimeric reads from short cfDNA fragments and validated the test using a control set of extracellular DNA samples obtained from pregnant women. The obtained sensitivity and specificity parameters of the NIPT developed by the authors corresponded to the approaches proposed earlier (99.93% and 99.14% for T21; 100% and 98.34% for T18; 100% and 99.17% for T13, respectively).

## 1. Introduction

NIPT (non-invasive prenatal testing) studies are carried out early in the pregnancy (starting at 9–12 weeks of gestation) to determine the presence of aneuploidy in the fetal genome. This test is done using freely circulating cell-free DNA (cfDNA) isolated from the mother’s blood, part of which is of placental origin [1]. Cell-free fetal DNA (cffDNA) enters the mother’s blood as a result of apoptosis of placental trophoblast cells and reaches a proportion of 4–15% by the end of the first trimester of pregnancy [2,3]. In the mother’s blood, cfDNA is in the form of fragments. Fragments of maternal cfDNA are predominantly 166 bp in length, and cffDNA fragments are predominantly 143 bp in length [4]. This distribution of the lengths is associated with non-random DNA cutting [5]. NIPT is a method for analysis of the relative number of copies of chromosomes in the extracellular DNA, and in most cases it is done using the shallow whole-genome sequencing (sWGS) approach. The obtained sequencing data of the corresponding chromosome normalized by the remaining chromosome coverages (chromosomal ratios) is compared to the results of analysis of the controlled set of samples with a known karyotype, using usually a *Z*-test. Reaching threshold values of *Z* statistics indicates the presence of aneuploidy on the corresponding chromosome in the fetus [6]. A threshold value is chosen in terms of a multiple of the chromosomal ratios’ standard deviation and generally is a three σ level (*Z*-score = 3) [7,8].

The proportion of fetal DNA is an important parameter, and as such is necessary to assess. If the values are too low, then the NIPT may result in a false negative. The two most common approaches of assessment are analysis of SNPs [4,6,9,10] and cfDNA length distribution [11,12]. SNP analysis is a deep sequencing (starting at 100×) of pre-amplified DNA fragments containing SNPs, according to which the mother and fetus may be differentiated using alleles inherited from the father. Analysis of several dozen such sites by counting reads containing paternal alleles determines the proportion of cffDNA. Length distribution analysis uses the results of paired-end (PE) mapped reads to obtain cfDNA length distribution data. The cffDNA proportion is estimated by comparing the number of reads of certain lengths, which is related to the difference in the initial length of the fetal and maternal cfDNA.

Next-generation sequencing (NGS) technologies compete in two areas: the number of molecules processed in a run and the read length, which reaches 200 and 250 bp in PE mode in modern sequencing platforms (MGISEQ-2000, MGI Tech Co., Shenzhen, China; NovaSeq 6000, Illumina, CA, USA). However, at a length as short as 40 bp, 82% of the molecules will be unambiguously mapped to the human genome [13]. Therefore, for applications not related to variant calling, increasing the length of the read does not bring additional benefits. More than 20 years ago, methods were developed to combine short DNA or cDNA fragments originating from different molecules into chimeric DNA molecules [14]. Thus, one chimeric molecule contains a set of tags that map to different parts of the genome. Chimeric molecules are extremely useful in studies requiring the determination of numbers of copies, such as differential gene expression studies or CNV searches in the genome [15].

**Figure 1:**
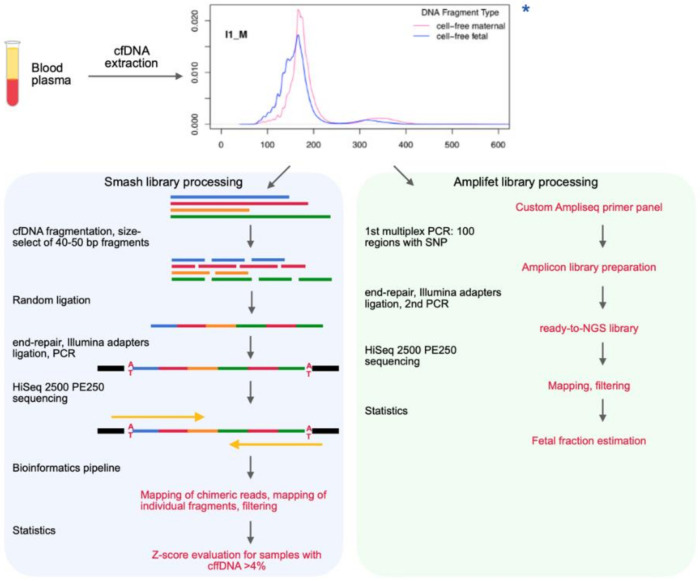
Schematic of NIPT (non-invasive prenatal testing) based on long-chimeric-read sequencing. Extracted cfDNA was divided into two pipelines in parallel: smash (blue) for aneuploidy evaluation and amlifet (green) for cffDNA fraction estimation. For the smash library, cfDNA was additionally fragmented to 40–50 bp fragments, and then was randomly ligated into long chimeric molecules. Whole chimeric reads were mapped to reference genome to identify each fragment’s ends. Fragments were then mapped as individual short reads. For the amplifet library, cfDNA was amplified with a primer pool to simultaneously obtain 100 amplicons with SNPs of interest. The amplicon library was then sequenced. An SNP was considered useful to estimate fetal fraction if maternal genotype was homozygous and fetal genotype was heterozygous. If fetal fraction >4%, aneuploidy statistical test was performed with smash data. * chart from Reference [16].

The goal of this work was to expand the laboratory and bioinformatics stages of NIPT using analysis of tags in chimeric molecules built from cfDNA fragments of pregnant women (Figure 1).

## 2. Materials and Methods

### 2.1. Ethics Statement

This study was conducted according to the principles expressed in the Declaration of Helsinki. Appropriate institutional review board approval for this study was obtained from the Ethics Committee at Pirogov Medical University (#170 at 18 December 2017). All patients provided written informed consent for the collection of samples, subsequent analysis, and publication thereof.

### 2.2. Sample Processing and Extraction of Blood Plasma and cfDNA

Pregnant women referred for invasive diagnostics at the Center for Family Planning and Reproduction in Moscow were recruited from December 2017 to December 2018. Maternal peripheral blood samples were collected into Streck BCT tubes just before obstetric procedures (such as chorionic villus sampling (CVS)) during the first trimester. Maternal peripheral blood samples were centrifuged at 250× *g* for 30 min, and then plasma was transferred to new 2.0 mL microtubes and centrifuged at 9000× *g* for 15 min. CfDNA was extracted using the QIAamp Blood kit (Qiagen, Hilden, Germany) with an increased volume of lysate transferred to the column (2–4 mL of blood plasma at the beginning) and volume of extraction decreased to 40 μL. QC was performed using the Qubit HS kit measuring on Qubit 2 (ThermoFisher, Waltham, MA, USA). A total 2 ng of cfDNA was used in the chimeric DNA library preparation and fetal fraction estimation stages of the assay.

To simplify, we refer herein to the two types of libraries used in our method as “smash” and “amplifet” libraries.

### 2.3. Chimeric DNA Library Preparation (“Smash”)

We refer to this type of library as “smash” in honor of the previously developed method [13], which prompted us to develop our methodology.

#### 2.3.1. Fragmentation

A total 2 ng of cfDNA was fragmented using the dsFragmentase kit (NEB, Ipswich, MA, USA): 1.25 μL of fragmentase reaction buffer v2, 2.5 μL of dsDNA fragmentase, 3 μL of 50% PEG-8000 solution (Sigma Aldrich, St. Louis, MO, USA), and MQ water to make up a total volume of 27 μL. The solution was incubated at 36 °C for 40 min.

#### 2.3.2. Double Size Selection to Obtain 40–50 bp Fragments

2× Ampure XP beads (Beckman, Brea, CA, USA) were added to 27 μL of fragmented products and incubated for 5 min at RT on benchtop. The microtube was then placed on a magnetic rack until all the beads were concentrated on one side of the microtube. Supernatant with DNA less than 100 bp in length was transferred to a new microtube. Lower size selection was done using QIAquick Nucleotide Removal Kit (Qiagen) to cut off DNA shorter than 40 bp. DNA was collected to make up a volume of 20 μL.

#### 2.3.3. End-Repair

End-repair reaction was done at RT for 180 min using a Quick blunting kit (NEB) by adding 2.5 μL of buffer, 2.5 μL dNTP mix, and 1 μL of enzyme mix.

#### 2.3.4. Self-Ligation

Formation of chimeric DNA molecules was done by adding 4.5 μL of T4 ligation buffer, 0.5 μL of T4 DNA ligase (E320 kit, Sybenzyme, Novosibirsk, Russia), 9 μL of 50% PEG-8000 solution, and 1 μL of 5’-deadenylase (NEB) to the product of the end-repair reaction. Self-ligation was conducted at RT overnight.

#### 2.3.5. A-Tailing

In this step, 4.5 μL of Taq buffer, 2 μL of Taq-pol (PK015L, Evrogen, Moscow, Russia), and 2 μL of dATP (R0181, ThermoFisher) were added to the self-ligation product to make up a final volume of 47 μL. The solution was incubated for 30 min at 65 °C and 2 min at 72 °C.

#### 2.3.6. Adapter Ligation

Oligonucleotides dir_1 (ACACTCTTTCCCTACACGACGCTCTTCCGATCT) and rev_P (Phos*GATCGGAAGAGCACACGTCTGAACTCCAGTC) were synthesized in Evrogen (Moscow, Russia). dir_1 and rev_P were diluted to 5 mM and combined in equal volumes, and then hybridized in a thermocycler by heating to 95 °C for 5 min and slow cooling to RT. To 47 μL of A-tailing product we added: 3 μL of adapter mix, 5.75 μL of ligation buffer, 3 μL of T4 DNA ligase (E320 kit, Sybenzyme, Novosibirsk, Russia), 6.3 μL of 50% PEG-8000 solution, and 0.5 μL of 5’-deadenylase. The reaction was incubated in a thermocycler for 100 cycles of the following program: 4 °C for 10 s, 16 °C for 30 s.

#### 2.3.7. Size Selection

1× volume of MQ water was added to the adapter ligation product, and then ×0.4 volumes of ×2 concentrated Ampure XP beads (Beckman, Brea, CA, USA) was added. Standard cleaning procedure was performed; however, DNA was not eluted from the beads. PCR mix from next step was added directly to the beads with immobilized DNA.

#### 2.3.8. Indexing PCR

In a new microtube, the following reagents were combined: 1 μL of i5 and 1 μL of i7 indexes from the E7600S NEB kit, 5 μL of HiFi buffer, 0.5 μL of HiFi pol, and 0.75 μL of dNTPs (all KAPA 7958897001). The mixture was added to beads with immobilized DNA and put into a thermocycler with the following program: 95 °C for 2 min; 98 °C for 20 s, 65 °C for 30 s, 72 °C for 2 min (for 15 cycles); 72 °C for 5 min.

#### 2.3.9. Cleanup

PCR products were cleaned with ×0.5 volume of Ampure XP beads. Elution was done in 20 μL of low TE.

#### 2.3.10. QC

**Figure 2:**
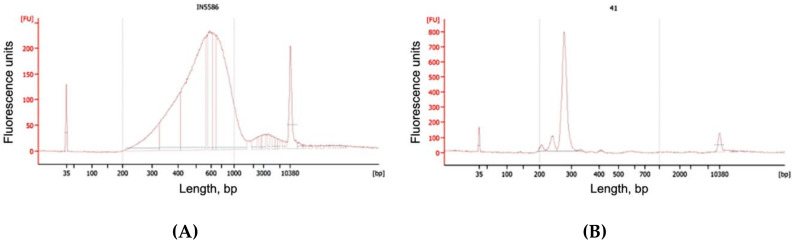
Electrophoregrams (Bioanalyzer 2100, Agilent) of chimeric DNA libraries: “smash” (**A**) and fetal fraction estimation library—“amplifet” (**B**).

QC was done using a high-sensitivity kit for the Bioanalyzer 2100 (Agilent, Santa Clara, CA, USA). Results were considered optimal if the library peak was in 500–800 bp range and the concentration was more than 4 nM in the 200–800 bp range (Figure 2A).

### 2.4. Choice of SNPs

SNPs were selected based on an analysis of 1000 Genomes [17] and gnomAD [18] databases. SNP filtering was performed using the VCFtools software package [19]. Markers in the human genome were selected according to the following criteria:

- Insertions, deletions, chromosomes X and Y, mitochondrial DNA and the p-arm of chromosome 6 (HLA region) were not included;
- Only diallelic polymorphisms were considered;
- Only SNP with identifiers “rs” were considered;
- Only alleles with a minimum allele frequency (MAF) of 0.4 were considered;
- We considered SNPs with a minimum probability of selection according to Hardy–Weinberg of p 0.00001 (i.e., the selection has almost no effect);
- Only unlinked SNPs were considered. They were determined using the sliding window method with the following parameters: correlation coefficient r2 < 0.5, sliding window size = 50 SNP, step = 5 SNP.

Next, only those SNPs that were common for samples from European populations were used for further analysis.

Primers were selected for the 610 selected SNPs using the AmpliSeq Designer software (www.ampliseq.com) with an amplicon size of no more than 140 bp. The nucleotide profile around the selected primers was analyzed and those that could have potential amplification problems (hairpins, homopolymers, etc.) were removed. Sequential filtering was done using the following criteria:

- absence of homopolymers in the primer (four or more identical nucleotides in a row);
- no repetitions (e.g., “ATATATA...”);
- absence of a large number of GC at the 3′ end;
- GC composition of the primer ranging from 0.4 to 0.6.

As a result, we designed a panel of primers for 102 SNPs, and ordered them to be synthetized from Thermo Fisher as a custom Ampliseq panel.

### 2.5. Fetal Fraction Estimation Library Preparation (“Amplifet”)

We refer to libraries for assessing the proportion of fetal DNA as “amplifet”. We used the standard approach to library construction from PCR products as the amplifet library preparation protocol: at the first stage, amplification of the regions of interest was carried out, and then adapters for sequencing were ligated to the purified amplicons with appropriately modified ends. We did not destroy the primer dimers using FuPa or mixtures containing uracilglycosylase, as they were effectively eliminated by the first washing with Ampure XP.

#### 2.5.1. Multiplex PCR

First, 2 ng of cfDNA was added to the first PCR with 20 μL of Amlpiseq primer mix (ThermoFisher), 8 μL of Phusion buffer, 0.4 μL of Phion U pol (F-555L kit, ThermoFisher), 0.8 μL of dNTPs (pb006L Evrogen), and MQ water up to 40 μL. The mixture was amplified using the following program: 98 °C for 30 s; 98 °C for 10 s, 60 °C for 4 min, 72 °C for 20 s for 27 cycles; 72 °C for 5 min.

#### 2.5.2. QC

Length of PCR products was assessed using agarose gel electrophoresis.

#### 2.5.3. Cleanup

The 1st PCR product was cleaned with ×3 volumes of Ampure XP beads and eluted to 20 μL.

#### 2.5.4. Adapter Ligation

To ligate Illumina DIY adapters (as in Section 2.3.6, described above), end-repair and A-tailing reactions were carried out in the same microtube but at a lower temperature. The following reagents were mixed in a new microtube: 10 μL of cleaned amplicons from 1st PCR, 5 μL of ligase buffer (B302 Sybenzyme), 5 μL of adapter mix, 0.5 μL of T4 DNA ligase (E330 Sybenzyme, Novosibirsk, Russia), 0.5 μL of 5′ deadenylase (M0331 NEB, Ipswich, MA, USA), 1 μL of 10 mM ATP (R0441 ThermoFisher, Waltham, MA, USA), 1 μL of Klenow exo- (m0212L NEB, Ipswich, MA, USA), 1 μL of dATP (R0141 ThermoFisher, Waltham, MA, USA), 2 μL of T4 PNK (EK0032 ThermoFisher, Waltham, MA, USA), 12 μL of 50% PEG-8000 solution, and 12 μL of MQ water. The mix was incubated at 37 °C for 40 min: 10 °C for 10 s, 30 °C for 30 s (100 cycles).

#### 2.5.5. Cleanup

The ligation product was cleaned with ×1.5 volumes of Ampure XP beads and eluted to 30 μL.

#### 2.5.6. Indexing PCR

The following reagents were mixed in a new microtube: 1 μL of i5 and 1 μL of i7 indexes from the E7600S NEB kit, 5 μL of Phusion buffer, 0.25 μL of Phusion U pol, and 0.5 μL of dNTPs. The mixture was put into a thermocycler and run through the following program: 98 °C for 30 s; 98 °C for 10 sec, 65 °C for 30 sec, 72 °C for 20 s (for 14 cycles); 72 °C for 5 min.

#### 2.5.7. Cleanup

Cleanup was done using a GeneRead Size Selection Kit (180514 Qiagen). DNA was eluted to 20 μL of Low TE.

#### 2.5.8. QC

QC was done using a high-sensitivity kit for the Bioanalyzer 2100 (Agilent). Results were considered optimal if the library peak was in the 270–280 bp range and the concentration was more than 4 nM in the 270–280 bp range (Figure 2B).

### 2.6. Sequencing

NGS was performed using an Illumina HiSeq 2500 instrument with Rapid Run v2 kits designed for 500 cycles (PE250 dual-indexing).

### 2.7. Bioinformatics

#### 2.7.1. Mapping

Raw data in BCL format were converted to FASTQ using bcl2fastq v. 2.20 software. Reads were mapped to the h38 genome in two iterations. Initially, BWA [20] version 0.7.17 with standard settings was used. In this case, smash-read fragments were distributed along the genome depending on where they were mapped. Reads with amplifet libraries were mapped entirely. During the second step, the subreads obtained as a result of the first mapping were extracted and mapped again as separate reads. Additional (supplementary) alignments and mapping onto the minus strand were filtered out (as after the first stage, all exported subreads had already been inverted into the plus strand of the reference genome).

#### 2.7.2. Filtering

For each smash library, the following steps were performed:

All BAM files that contained smash data for the sample were downloaded and, if there were several, were combined using SAMtools [21] version 1.9.

The following reads were filtered out:

- those that were imperfectly mapped onto the genome (MAPQ < 60);
- those that fell into regions of known repeats (RepeatMasker in Genome Browser track);
- those that fell into amplicon regions.

For each amplifet library, only reads that fell into the amplicon region (off-target reads were depleted) were filtered out.

The FLASH tool [22] with the following settings was used to calculate insertion length in smash libraries:

—min-overlap 20—max-overlap 250—allow-outies—max-mismatch density 0.20.

### 2.8. Statistics

We ran FetalQuant [23] with the default parameters to estimate the fractional fetal DNA concentration from the SNP data (amplifet target sequencing). The training samples were classified as case/control based on chromosomal *Z*-scores. We used the R-package NIPTer [24] to perform a variation reduction (peak, GC, and chi-squared corrections), match QC, and calculate the *Z*-score.

#### Filtering

We filtered out all samples in which the estimated fractional fetal DNA concentration was less than 4% or the total number of fragments was less than 2,500,000. We used a threshold of 3 for the *Z*-score in the classification task. To make the estimates of the accuracy characteristics more stable, we implemented a kind of cross-validation procedure. The test dataset was composed of all samples with trisomies (for a certain chromosome) and an equal number of the control samples chosen randomly. The remaining control samples formed the training dataset. For each run we computed sensitivity, specificity, and area under curve (AUC) values for the classification task and reported the averaged values over 200 runs.

### 2.9. Determination of the Sex of the Fetus

To determine the sex, a formula for estimating the fraction of fetal DNA was used based on the analysis of the fraction of fragments of the Y chromosome [25,26,27].

## 3. Results

### 3.1. Clinical Characteristics

The study included 145 women aged 20 to 48 years. The gestational ages varied from 11 to 25 weeks (15.1 weeks on average). Demographic and clinical characteristics of the patients are presented in Table 1. All subjects had a singleton pregnancy, were not carrying a donor egg, did not have clinically established rearrangements in their genome, had not gone through an embryo reduction procedure, were not cancer patients, had not undergone blood transfusions during the six months prior to the biomaterial collection procedure, and also had not undergone organ transplantation, including bone marrow or stem cell therapy.

### 3.2. Extraction of cfDNA

We used the QIAamp DNA Blood Mini Kit to isolate cfDNA from 3–4 mL of blood plasma as previously described in Reference [28].

### 3.3. Smash Protocol

The library preparation protocol we propose differs from the classical NIPT protocol in three elements:

1. Fragmentation of cfDNA to short fragments;
2. Size selection of the fragmented cfDNA from two sides, leading to a number of fragments with an average length of 40–50 bp remaining in the sample;
3. The random ligation of short cfDNA fragments and thereby the formation of long (more than 300 bp) chimeric DNA molecules to which adapters for NGS are already ligated.

**Figure 3:**
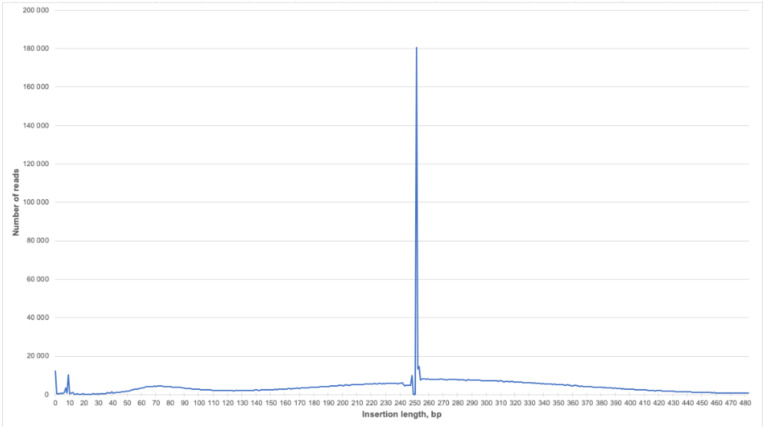
Insertion length distribution for PE reads of the smash library. The X axis is the insertion length in bp, and the Y axis is the number of reads of the corresponding length.

We were able to obtain a larger amount of useful diagnostic information from each read compared to the classic NIPT because of the selected sample preparation conditions and long-read reagents (Rapid Run PE250). The average fragment size in the test samples was 43.8 bp. Thus, the information content of one direct or reverse read with a length of 250 bases was 5.7 times higher than for the classic single read. Since it was impossible to determine the size of the insertion of a pair of reads by mapping them onto the reference genome, in our case we tried to estimate the initial sizes of sequenced chimeric molecules by fusing them using the FLASH tool. FLASH was developed to find the correct overlap between paired-end reads and extend the reads by stitching them together. If the fragments are shorter than twice the read length, the resulting paired-end reads will overlap. The length distribution of chimeric molecules for one of the typical samples is shown in Figure 3. It can be seen that most of the reads had a length of about 250 bp. This means that it was not possible to fuse the pairs of reads, and the length of the chimeric molecule for them exceeded 460 bp, which correlated with the histogram data from the Bioanalyzer (Figure 2A).

### 3.4. Smash Sequencing Results

The sequencing results for a subset of smash libraries are shown in Supplementary Table S1 of Supplementary Files. By “filtered fragments”, we mean subreads from chimeric long reads that passed the filtration stages described in the corresponding section of Materials and Methods. The total length of the regions in the RepeatMasker track was 1,586,326,530 nucleotides, which is approximately half the human genome; therefore, it can be assumed that the total number of fragments per chimeric read before filtering was twice that after filtering. Chimeric reads were read using the paired-end method for 250 nucleotides in both directions. Every pair of 250 bp reads contained 2.37 filtered fragments. It was approximately 4.6 times higher than a classic NIPT pair, because half of them were filtered during application of the RepeatMasker.

### 3.5. Amplifet Sample Sequencing Results

**Figure 4:**
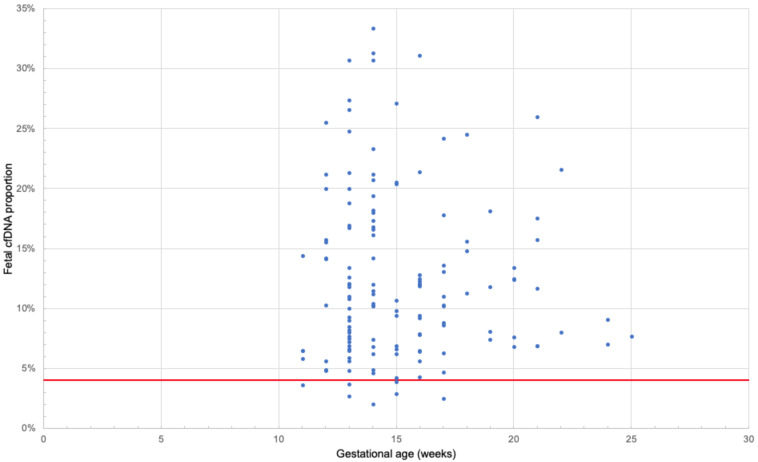
The proportion of fetal DNA (Y axis) versus the gestational age of 139 samples of the control set (X axis), calculated based on the results of amplifet library sequencing. The solid red line indicates a 4% proportion of fetal DNA.

Amplifet library sample sequencing results are shown in Supplementary Table S2 of Supplementary Files. In the control subset of samples, 96 out of 102 systems worked (94%). On average, 5099 reads were obtained for each point. For two samples (za6077, zi2425), the average coverage at the point was less than 50×; therefore, they were excluded from further analysis because they provided no reliable assessment of the proportion of fetal DNA. SNPs for which the fetus genotype was heterozygous and the mother was homozygous were considered informative. Thus, the proportion of fetal DNA was defined as the fraction of reads per paternal allele doubled. On average, the proportion of fetal DNA in the set of the control samples was 12%, which is consistent with the results obtained for this range of gestational ages [29] (Figure 4). Four samples were excluded from further analysis as it was not possible to confirm data on gestational dates for them from the clinic (jt4805, ds3710, in5586, gv2133).

### 3.6. Determination of the Minimum Number of Fragments in the Sample

**Figure 5:**
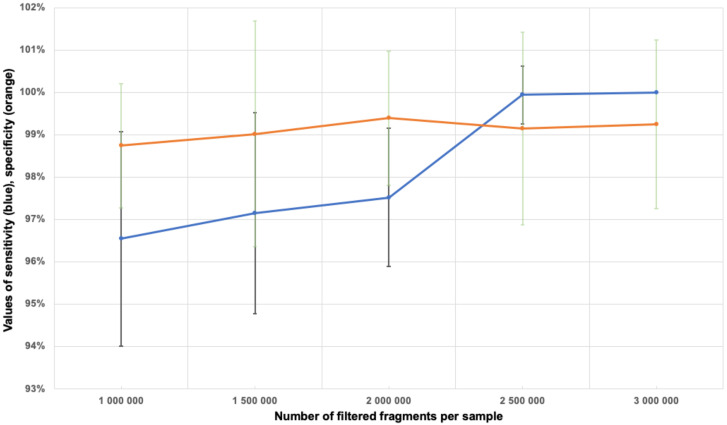
The sensitivity (blue line) and specificity (orange line) values of the determination of aneuploidy on Chromosome 21 with regard to the standard deviation, depending on the number of filtered fragments per sample.

We determined how many fragments that passed through the filters were necessary and sufficient to determine aneuploidy. This was done by calculating the sensitivity and specificity of aneuploidy determination on Chromosomes 21, 18 and 13 for samples with 1 to 3 million filtered fragments in increments of 0.5 million fragments. The calculation results are shown in Table 2. Figure 5 shows the specificity and sensitivity values calculated for aneuploidy of Chromosome 21. Thus, a minimum of 2.5 million filtered fragments per sample was determined to be optimal.

### 3.7. Sample Filtration

Samples were filtered in accordance with the criteria for the minimum number of filtered fragments defined above, as well as a cutoff value for the minimum fraction of fetal DNA in the sample of 4% [30]. A total of 83 samples were included in the final sampling (normal karyotype—48 samples, 58%; T21—25 samples, 30%; T18—6 samples, 7%; T13—4 samples, 5%). The list of final samples and their characteristics is presented in Supplementary Table S3 of Supplementary Files.

### 3.8. Sensitivity and Specificity Assessment for Aneuploidy

**Figure 6:**
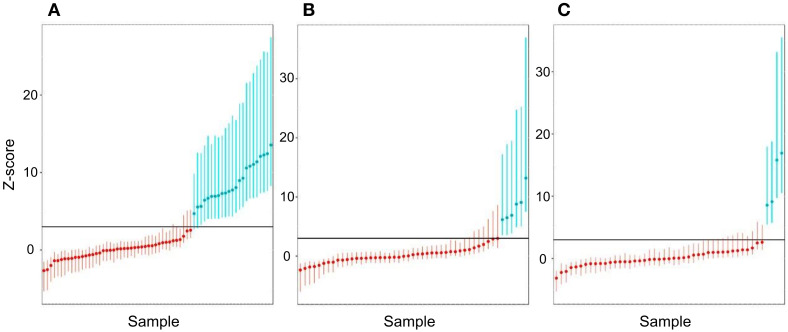
Histograms of *Z*-score values obtained during the cross-validation of control samples ((**A**)—T21, (**B**)—T18, (**C**)—T13 chromosome). Red indicates samples that did not have the corresponding aneuploidy, green indicates samples with aneuploidy. The maximum and minimum *Z* values for the sample in the simulations are plotted by error bars. SD values are given in Supplementary Table S4 of Supplementary Files. The black horizontal line in the histograms indicates *Z* = 3.

To assess the sensitivity and specificity of the test, we used a *Z*-score threshold of 3. To increase the sample size in the process of obtaining robust estimates of sensitivity, specificity, and AUC, a cross-validation procedure was used, described above in the Materials and Methods. The results of the *Z*-score calculation for the corresponding trisomies are shown in Figure 6, as well as in Supplementary Table S4 of Supplementary Files.

Results of the sensitivity and specificity assessment for aneuploidy are presented in Table 3. Our results were consistent with the values obtained by other researchers [31,32,33].

### 3.9. Calculation of PPV and NPV for Aneuploidy

Positive predictive value (PPV) and negative predictive value (NPV) are operational characteristics of the test, and take into account not only the sensitivity and specificity metrics of our methodology, but also the disease frequency in the population. Since our test can be used in populations with different trisomy frequencies (for example, in at-risk groups according to the results of the first trimester screening), we calculated PPV and NPV for the corresponding frequencies of aneuploidy. The results are shown in Table 4. Correct interpretation of the results should be based on trisomy prevalence in the population: 22.0 (95% CI 21.7–22.4) for T21, 5.0 (95% CI 4.8–5.1) for T18 and 2.0 (95% CI 1.9–2.2) for T13 per 10,000 births [34].

### 3.10. Determination of the Sex of the Fetus

**Figure 7:**
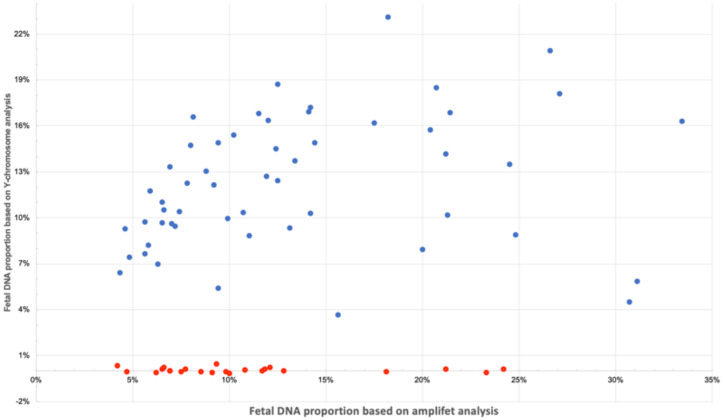
Fetal DNA proportion calculated for amplifet (X axis) and Y chromosome (Y axis). Male samples are indicated in blue, female samples in red.

Determination of the sex of the fetus was carried out using calculations of the normalized coverage of the Y chromosome. As can be seen in Figure 7 and in Supplementary Table S5 in Supplementary Files, male and female fetuses were well divided into groups. If the proportion of fetal DNA exceeded 4% and the proportion of the Y chromosome was not higher than 1%, the fetus was female. It can be seen that the results of the determination of the fetal DNA proportion in male samples for amplifet and the Y chromosome tended to correlate. We predict that further adjustments to the Y chromosome calculation system could closely correlate with other methods for determining the proportion of fetal DNA. It is also possible that a similar approach of comparing X chromosomes and autosome coverages could be used to determine the proportion of fetal DNA in mothers pregnant with females.

## 4. Discussion

In this study, we showed the possibility of using long chimeric reads for NIPT in a control set of 83 extracellular DNA samples from pregnant women with different fetal karyotypes. Our results showed that the use of the combined approach (smash and amplifet libraries) can make better use of modern massive parallel sequencing technologies that use long reads. This is especially relevant if we consider the trend of increasing read lengths in recent years (for example, single-end 400 bp on MGISEQ-2000 or extra-long reads in nanopore sequencing technology). Our technique can be easily adapted for use with Oxford Nanopore technology. A similar study was conducted by our colleagues for CNV analysis [35]. With a good training set of cfDNA samples from pregnant women, the possibility of NIPT using nanopore sequencers is promising [36].

Despite the obvious advantages of using NIPT in clinical practice, the widespread introduction of the test is largely limited by the high cost of its implementation. Our calculation of net cost per sample was $122 (from blood sample collection to report). For comparison, Illumina’s VeriSeq NIPT Solution v2 kit costs $200 per sample [37]. The advantages of VeriSeq include automated library preparation, cffDNA fraction estimation based on cfDNA length, and shorter turnaround time (day workflow). The long-chimeric-read technique could be automated too. The possibility of direct cffDNA fraction estimation from long chimeric reads should be estimated in additional research on a larger set of samples. Sample processing time could decrease if newer sequencers were to be used. The technique demonstrated in this publication could significantly reduce the cost of NIPT, thereby making it more accessible to patients. Of course, this requires more extensive studies of the clinical efficacy of our test on larger patient samples.

It is important to note that we did not include an analysis for sex chromosome abnormalities, such as Turner or Klinefelter syndrome, because no samples with sex chromosome pathologies were presented in the control sample set. We also did not estimate the reliability of our technique on typical confounding cases for classic NIPT; namely multiple gestations, vanishing twin syndrome, and diagnosis of cancer in the mother. Additional research should be conducted on a larger set of samples. In our study, we proved the possibility of using long chimeric reads in NIPT.

We also see promise in the technique for estimating the proportion of fetal DNA directly from sequencing data of smash libraries. This will simplify the sample preparation procedure and reduce its cost. Despite the use of additional fragmentation, in which we lose information about the initial length of a particular cfDNA molecule, the ratio of extracellular DNA, as well as its 5’and 3’ ends, is preserved in the fetus and mother. Therefore, with an increase in the size of the training set, it will become possible to use methods to analyze the nucleosome profile of the ends of the fragments [38]. Another approach may be the use of neural networks [39]. This approach is also limited by the size of the training sample set, so, in the future, we plan to conduct additional research on a larger set of samples.

## 5. Conclusions

In conclusion, we developed a novel variant of NIPT and demonstrated the validity of the approach based on long chimeric reads. This technology already seems more economically justified for routine laboratory practice; however, there are obvious directions in which our approach should be improved. In particular, it is important to develop alternative methods for assessment of the proportion of fetal DNA.

## Figures and Tables

**Table 1:** Clinical and demographic characteristics of the sampled patients.

Characteristic	Value
Number of patients	145
Average age (range) (years)	35.6 (20–48)
Mean gestational age (weeks)	15 (11–25)
Median gestational age (weeks)	14
Median weight of the pregnant women (range) (kg)	65 (47–108)
Median height of the pregnant women (range) (cm)	165 (150–185)
**Race or ethnic group, number (proportion in %)**	
white	138 (95.2)
of which Slavonian	131 (94.9)
Asian	7 (4.8)
not indicated	7 (4.8)
**Result of the invasive diagnostic, number (proportion in %)**	
normal karyotype	82 (56.6)
chromosome trisomy 13	4 (2.8)
chromosome trisomy 18	14 (9.7)
chromosome trisomy 21	45 (31)

**Table 2:** Sensitivity and specificity calculation of the test for samples with the corresponding minimum number of unique fragments.

Chromosome	Minimal Filtered Fragment Threshold	Sensitivity	Sensitivity SD	Specificity	Specificity SD
13	1,000,000	1	0	0.99536	0.011605
13	1,500,000	1	0	0.98883	0.030454
13	2,000,000	1	0	0.9952	0.020135
13	2,500,000	1	0	0.99169	0.032054
13	3,000,000	1	0	0.99556	0.012202
18	1,000,000	1	0	0.98154	0.023683
18	1,500,000	1	0	0.97256	0.04133
18	2,000,000	1	0	0.98368	0.020579
18	2,500,000	1	0	0.98342	0.031326
18	3,000,000	1	0	0.9925	0.01991
21	1,000,000	0.96537	0.025367	0.98735	0.014686
21	1,500,000	0.97143	0.023793	0.99017	0.026724
21	2,000,000	0.97517	0.016273	0.99388	0.015848
21	2,500,000	0.99935	0.0068607	0.9914	0.022761
21	3,000,000	1	0	0.9925	0.01991

**Table 3:** Sensitivity and specificity of the method for the sample set. Confidence interval was determined based on Wilson’s score method.

	Trisomy 21	Trisomy 18	Trisomy 13
**Sensitivity**	99.93%	100%	100%
**2-sided 95% CI**	(85.58–99.99%)	(60.96–100%)	(51.01–100%)
**Specificity**	99.14%	98.34%	99.17%
**2-sided 95% CI**	(90.29–99.93%)	(91.16–99.7%)	(92.7–99.91%)

**Table 4:** Positive predictive value and negative predictive value of our NIPT assay for detecting trisomies 21, 18, and 13 for a range of prevalences.

Aneuploidy	Prevalence	PPV	NPV
**Trisomy 21**	0.05%	5.491%	100.000%
	0.10%	10.415%	100.000%
	0.20%	18.880%	100.000%
	0.50%	36.853%	100.000%
	1.00%	53.983%	99.999%
	1.50%	63.881%	99.999%
	2.00%	70.328%	99.999%
**Trisomy 18**	0.03%	1.777%	100.000%
	0.05%	2.928%	100.000%
	0.10%	5.693%	100.000%
	0.20%	10.782%	100.000%
	0.30%	15.358%	100.000%
	0.40%	19.496%	100.000%
	0.50%	23.255%	100.000%
**Trisomy 13**	0.01%	1.190%	100.000%
	0.02%	2.352%	100.000%
	0.05%	5.680%	100.000%
	0.10%	10.755%	100.000%
	0.20%	19.437%	100.000%

## Supplementary Materials

The following are available online at https://www.mdpi.com/2073-4425/11/6/590/s1, Table S1: sequencing metrics for a subset of smash libraries. Table S2: sequencing metrics for a subset of amplifet libraries. Table S3: list of control samples passed through filtering. Table S4: *Z*-score values obtained during the cross-validation of control samples. Table S5: determination of the sex of the fetus.
